# CHESS (CgHExpreSS): A comprehensive analysis tool for the analysis of genomic alterations and their effects on the expression profile of the genome

**DOI:** 10.1186/1471-2105-10-424

**Published:** 2009-12-16

**Authors:** Mikyung Lee, Yangseok Kim

**Affiliations:** 1 Department of Physiology, College of Oriental Medicine, KyungHee University, #1 Hoegi-Dong, Dongdaemun-gu, Seoul 130-701, South Korea

## Abstract

**Background:**

Genomic alterations frequently occur in many cancer patients and play important mechanistic roles in the pathogenesis of cancer. Furthermore, they can modify the expression level of genes due to altered copy number in the corresponding region of the chromosome. An accumulating body of evidence supports the possibility that strong genome-wide correlation exists between DNA content and gene expression. Therefore, more comprehensive analysis is needed to quantify the relationship between genomic alteration and gene expression. A well-designed bioinformatics tool is essential to perform this kind of integrative analysis. A few programs have already been introduced for integrative analysis. However, there are many limitations in their performance of comprehensive integrated analysis using published software because of limitations in implemented algorithms and visualization modules.

**Results:**

To address this issue, we have implemented the Java-based program CHESS to allow integrative analysis of two experimental data sets: genomic alteration and genome-wide expression profile. CHESS is composed of a genomic alteration analysis module and an integrative analysis module. The genomic alteration analysis module detects genomic alteration by applying a threshold based method or SW-ARRAY algorithm and investigates whether the detected alteration is phenotype specific or not. On the other hand, the integrative analysis module measures the genomic alteration's influence on gene expression. It is divided into two separate parts. The first part calculates overall correlation between comparative genomic hybridization ratio and gene expression level by applying following three statistical methods: simple linear regression, Spearman rank correlation and Pearson's correlation. In the second part, CHESS detects the genes that are differentially expressed according to the genomic alteration pattern with three alternative statistical approaches: Student's t-test, Fisher's exact test and Chi square test. By successive operations of two modules, users can clarify how gene expression levels are affected by the phenotype specific genomic alterations. As CHESS was developed in both Java application and web environments, it can be run on a web browser or a local machine. It also supports all experimental platforms if a properly formatted text file is provided to include the chromosomal position of probes and their gene identifiers.

**Conclusions:**

CHESS is a user-friendly tool for investigating disease specific genomic alterations and quantitative relationships between those genomic alterations and genome-wide gene expression profiling.

## Background

It is well-known that genomic alterations frequently occur in many cancer patients and play important mechanistic roles in pathogenesis of cancer. Recently, in order to identify genomic alteration regions, a comparative genomic hybridization (CGH) technology has been extensively applied to various types of cancer cases. CGH is a molecular cytogenetic method that detects gain or loss of genomic DNA content of an individual, which is accomplished by measuring the ratio between the intensity of test DNA and that of reference DNA [1]. As the technology of array-based CGH has advanced, the resulting unprecedented detailed examination of chromosomal regions has led to efforts to discover genomic alterations as the genetic markers for various diseases [2,3]. Those efforts have served to emphasize the fact that genomic alterations play important roles in a particular disease. Furthermore, genomic alterations can modify the expression level of genes due to changed copy number in the relevant chromosomal regions. Recent studies have been concerned with verifying the existence of a strong genome-wide correlation between DNA content and gene expression [4,5]. These important CGH-based biological discoveries have spurred the widespread use of the technique, which has prompted the need for a genomic alteration analysis tool.

Accordingly, statistical algorithms and programs such as GEAR, aCGHViewer, and CAPweb have been developed to analyze and visualize CGH data. However, these programs have concentrated on analyzing genomic alteration and do not deal with an integrative analysis of genomic alteration and gene expression [6-8]. A few non commercial programs have been introduced for integrative analysis such as ACE-it, ISACGH, VAMP, and SIGMA^2 ^[9-12]. While ACE-it and ISACGH are focused on the analysis of genomic CGH and gene expression, VAMP and SIGMA^2 ^are implemented to deal with various genomic molecular profiles. Those programs are well implemented according to their analysis goals. However, they have limited algorithms and visualization modules, it is not easy to perform comprehensive analysis of integrated analysis of genomic CGH and gene expression. Table 1 compares the features of the introduced programs, the number in parenthesis means the number of offered statistical methods. Additionally, some programs need the non familiar package like a R. Therefore, we have developed an integrative analysis tool named CHESS to allow comprehensive analysis of genomic CGH data and gene expression data within an easy-to-use single consistent interface using a variety of statistical methods. CHESS is composed of two successive modules: a genomic alteration analysis module and an integrative analysis module of genomic alteration and gene expression. The complete successive operations of these two modules provide two kinds of biologically meaningful information: phenotype or class specific genomic alteration regions, and a list of genes whose expressions are affected by those genomic alteration regions. CHESS supports all experimental platforms if a properly formatted text file is provided to include chromosomal position and their gene symbol of probes. We presently provide information concerning CHESS. A detailed description of the entire procedure and file format is provided in the online manual; http://biostone.khu.ac.kr/CHESS/.

**Table 1:** CHESS's main features

Features	ACE-it	ISACGH	VAMP	SIGMA^2^	CGH Analytics	CHESS
Built-in GA detection		✓	✓	✓	✓	✓
Frequent GA region definition		✓	✓	✓	✓	✓
Case/control study				✓		✓
Enrichment analysis for GO and KEGG		✓				✓
Integrative analysis of genomic CGH and gene expression	✓(1)	✓(2)	✓(2)	✓(3)	✓(2)	✓(6)
Free for academic	✓	✓	✓	✓	✓	✓
Web accessible		✓	✓			✓

The number in parenthesis means the number of offered statistical methods

## Implementation

We chose Java as the programming language because it is publicly available and ensures cross-platform compatibility. Moreover, CHESS was developed in both Java application and webstart environments; it can be run on a web browser or a local machine. If CHESS is operated through a web browser, any uploaded data is not transmitted anywhere because all the analysis are performed locally using webstart. CHESS can deal with high-density arrays on commonly used desktop computers. For example, we were able to load 30 Agilent 244 k arrays in 3 min on a computer with 3 GB of memory and a 2.4 Ghz processor.

## Results

### Analysis flow

CHESS is composed of two primary modules: a genomic alteration analysis module and an integrative analysis module. The first module is responsible for the detection of genomic alteration regions and investigates whether the detected regions are phenotype specific or not. Genes located in the altered regions are automatically listed and biological information is given by an implemented annotation module. The integrative analysis module includes a combined analysis of genomic alteration and gene expression. For user's rapid understanding of the complete analyzed results, CHESS provides a resulting figure on a whole chromosomal scale. CHESS also provides the analyzed results on a single chromosomal scale for further detailed analysis.

### Data Requirements

CHESS was developed to support all experimental platforms if only a properly formatted text file is given including general information such as probe identification, chromosomal location and normalized log transformed ratio values for each probe. CHESS handles two sets of experimental data: signal ratio values from a CGH experiment and those from a gene expression experiment. Firstly, the CGH ratio file acquired from the CGH experiment should define header information in the first four lines; total number of samples, total number of probes, sample names and their clinical information. The test/reference ratio values should be written from the fourth line, in which the first five fields record probe identification, probe name or alias, chromosome number, start position and stop position with tab separations. The rest of the columns are considered as the ratio values for each sample. When the user successfully loads the CGH ratio file and finishes detecting the genomic alteration regions, a GA (Genomic Alteration) file is automatically generated that has the exactly the same format as the CGH ratio file. However, the GA file contains discrete values concerning genomic alteration in the form of gain (+1), loss (-1) and no genomic alteration (0), instead of the continuous signal values of the CGH ratio file. To support the output from other detection algorithms as aCGH [13] and DNAcopy [14], CHESS allows direct loading of the GA file. Secondly, the gene expression file is also a tab delimited text file to record the level of mRNA expression. It can have two kinds of data formats according to its experimental method, single channel array and dual channel array. The single channel array hybridizes two samples on the separate arrays and makes two separate files that record intensity values for test and reference samples. In the other hand, the dual channel array hybridizes two samples on the same array and creates one ratio file that records ratio values of test/reference intensity values. CHESS handles these two kinds of data formats and they have same file format. The first two lines include information on data dimension of used sample number and probe number. The third line lists the sample names separated by tabs. The rest of the lines record ratio values or intensity values for entire probe in which the first three columns list probe name, the corresponding gene symbol, chromosome number and the subsequent columns record actual expression values. Finally, the gene mapping file is needed to match probes used in CGH to a corresponding gene for biological interpretation. It has a very simple format, in which the gene symbol is followed by CGH probe identification with tab separations.

### Definition of genomic alteration

**Figure 1:**
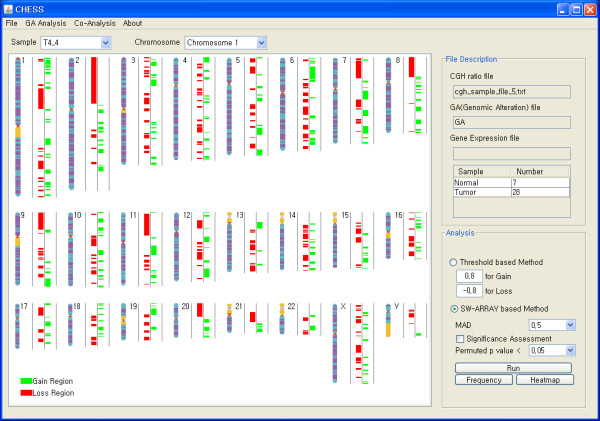
**Screenshot of detection of genomic alteration region**. After detection of genomic alterations, CHESS displays graphically detected altered regions for each sample on the whole chromosomal mode, in which green and red indicate an amplified or deleted region, respectively. Single chromosomal view can be shown by selecting the chromosome of interest in a drop-down list.

**Figure 2:**
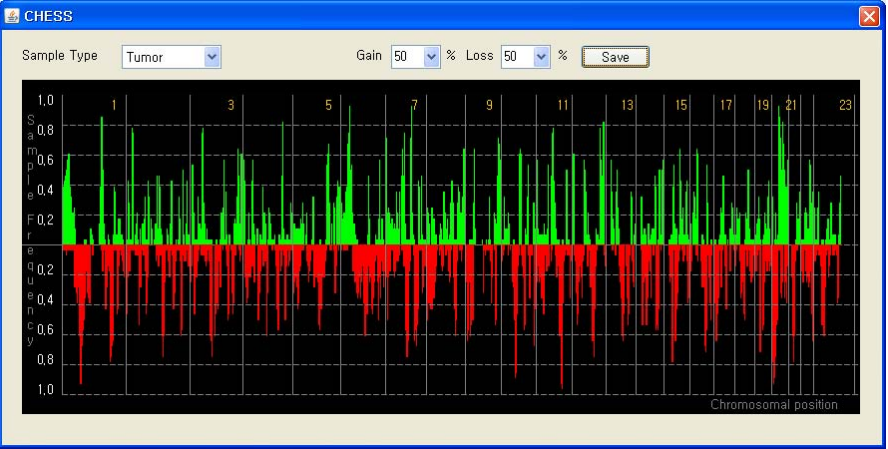
**Sample frequency display**. After detection of genomic alterations, sample frequency plot is drawn in a separate panel to help users observe the genomic alteration pattern across whole samples in a straightforward manner.

CHESS defines genomic alteration regions with two distinct ways: user-specified threshold and SW-ARRAY-based algorithm. The threshold based method defines individual probe as gain or loss if the ratio value is higher or lower than user specified threshold, respectively. CHESS also has the widely used SW-ARRAY algorithm that is known to be a robust and reliable method for detection of genomic alterations [15]. It determines locally high-scoring segments whose score cannot be increased by shrinking or expanding the segment boundaries by applying Smith-Waterman dynamic-programming algorithm. After detecting genomic alterations, a GA file is automatically generated to record information regarding the alterations in the form of gain (+1), loss (-1) and no genomic alteration (0). After detection of genomic alterations is completed, CHESS shows these altered genomic regions in the form of a color bar, in which green and red indicate the regions on each chromosome that are amplified and deleted, respectively (Figure 1). A detailed view on the single chromosomal scale can be generated by selecting the chromosome of interest in a drop-down list. Sample frequency plot and heatmap drawn in a separate panel intuitively assists users in observing the overall genomic alteration pattern across whole samples intuitively (Figure 2).

### Identification of phenotype specific genomic alteration regions

**Figure 3:**
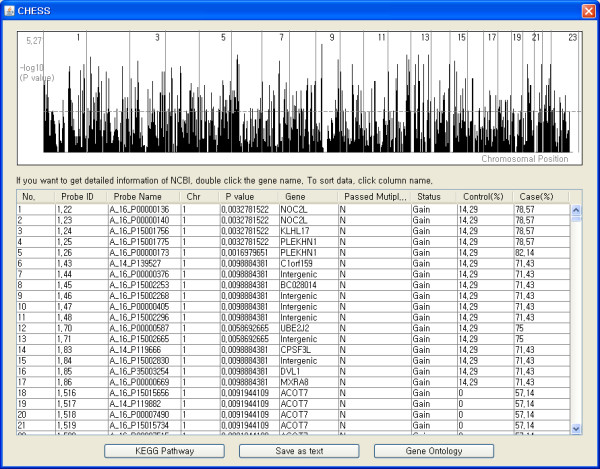
**Association study display**. The panel on the top displays the log transformed p values as a function of chromosomal location. The table below summarizes the probes determined to be significant for discriminating between case and control types. Double clicking the interesting gene name leads user to the web page of NCBI's Entrez gene.

CHESS identifies the phenotype or class specific genomic alteration regions based on the given clinical information. For this association study, continuous raw signal ratio values or discrete values of genomic alteration status are needed as essential information. When continuous signal ratio values are read, the one-sided Student's t-test can be applied to investigate the null hypothesis that the ratio values of chosen control and case types have the same distribution in their CGH ratios. If the GA file is generated and the information about genomic alteration status is available, users can carry out Fisher's exact test or Chi-square test. When sample size is relatively small, Fisher's exact test is recommended because it calculates exactly the significance rather than assuming approximated distribution like a Chi-square test. After the association study between case and control is completed, the probes determined to be significant in distinguishing between case and control types are summarized with their p values and corresponding gene symbol in a separate panel (Figure 3). To compensate for multiple test of association study, the adjusted result by Bonferroni correction will be also presented. When the association study is finished, CHESS marks the genomic regions including the significant probes as a green/red bar on the whole chromosomal scale. A single chromosomal panel plots the log transformed p value for entire probes according to chromosomal locations. Finally for biological interpretation, CHESS gives the significance value calculated from hypergeometric distribution to represent enrichment for KEGG pathways and Gene Ontology in which phenotype specific genomic regions are involved.

### Integrative analysis of genomic alteration and gene expression data

**Figure 4:**
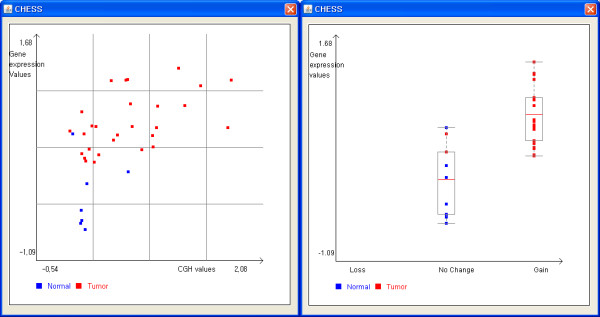
**Scatter plot of CGH ratio and gene expression values**. The scatter plot graphically expresses CGH ratios and gene expression values in horizontal and vertical axes, respectively. It is useful for investigating the influence of genomic alteration on gene expression level in an intuitive fashion.

Integrative analysis module of CHESS can be divided into two separate parts. The first part calculates the overall correlation values between CGH ratio values and gene expression level. The second part determines genes whose expression patterns are significantly different according to the status of genomic alterations. For correlation analysis, CHESS provides three statistical methods: simple linear regression, Spearman rank correlation and Pearson's correlation. These calculate correlation values for all possible pairs of CGH probes and gene expression probes mapped to the same gene. While simple linear regression calculates the r^2 ^with real ratio values, Spearman rank analysis calculates the ρ (Spearman's rho) using their corresponding rank values instead of real ratios. When the correlation analysis is finished, the obtained correlation values can be saved in a new text file and are indicated in the form of colour bars on both the whole chromosomal and single chromosomal scales. The length and colour of the bar corresponds to the size and the direction of correlation, respectively. The scatter plot between CGH ratio values and gene expression values is also given to users for the intuitive understanding of the general correlation pattern (Figure 4). In addition to correlation analysis, CHESS determines genes whose expression patterns are significantly affected by their genomic alteration status through three statistical methods: One sided Student's t-test, Fisher's exact test and Chi square test. Student's t-test examines the null hypothesis that the gene expression values have the same distribution regardless of genomic alteration pattern. Fisher's exact test and Chi square test investigate whether gene expression level is affected by genomic alteration after defining the status of gene expression (up, down and no change) under the user-specified threshold. These two tests are carried out twice to test the hypothesis that the amplified genomic region leads to a significant up-regulation of mRNA and the deleted region leads to a significant down-regulation of mRNA. After this analysis, the plot to express the gene expression values and genomic alteration status in vertical and horizontal axes, respectively, is presented in a separate panel (Figure 4).

### Case study of CHESS using colorectal cancer data set

**Figure 5:**
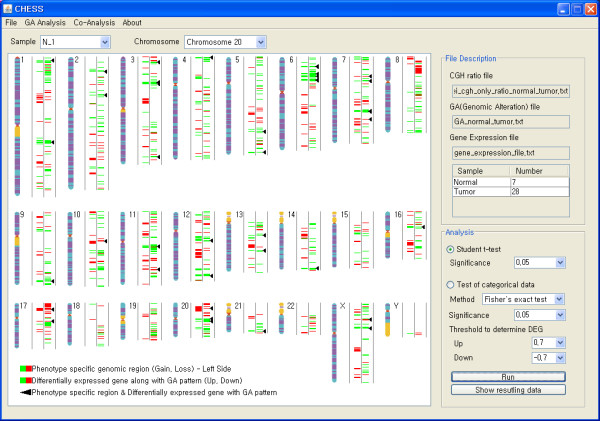
**Whole chromosomal panel after performing genomic alteration and integrative analyses**. In this whole chromosomal panel, genomic regions identified as colorectal cancer specific regions by Fisher's exact test (p < 0.0001) are colored green (gain) and red (loss) in the left of bar. The red and green colors in the right of bar indicate genes whose expression patterns are significantly different according to the genomic alteration patterns (Student's t-test, p < 0.01). The black arrows represent genes whose expression levels are affected by colorectal cancer specific genomic alteration regions.

To illustrate the usability of CHESS, we have used our own colorectal cancer data set. The data set consists of genomic CGH data from Agilent 244 k CGH arrays and 17 k cDNA gene expression data for 7 normal and 28 colorectal cancer patient samples. To predict the genes whose expression levels are significantly affected by genomic alteration, we have performed two kinds of statistical methods, Spearman rank correlation and student t-test. As the result of analysis, 565 and 300 genes were identified from Spearman rank correlation (ρ > 0.5) and student t-test (*P *< 0.01) respectively. We have compared gene lists from each analysis, and found only 76 genes are common in both analyses. The different result from each analysis may be due to the characteristics of each method. The correlation based methods like a Spearman rank correlation and Pearson correlation coefficient are more sensitive than the categorical data analysis methods such as student t-test, Fisher's exact test and chi-square test. However, it cannot reflect biological phenomena that genomic alteration has a discrete nature. Therefore, each analysis method has its own strength and weakness, it is very important for users to attempt various statistical methods and to interpret the biological meaning in various angles. Among 76 common genes, BCL2L1 known to be the anti-apoptotic gene has shown a clear correlation between genomic alteration and expression pattern (Figure. 4). Additionally, figure 5 shows the analysis result after conducting two successive modules (genomic alteration analysis, integrative analysis). The red/green in the left side of a bar represents the colorectal cancer specific genomic alteration regions (loss/gain). The red/green of the right side means the significantly differentially expressed genes according to the genomic alteration pattern. The black arrows represent genes whose expression levels are affected by colorectal cancer specific genomic alteration regions.

## Conclusions

We have implemented a Java-based program named CHESS for the comprehensive analysis of genomic alteration. Functionally, CHESS can be divided into two parts. The first function is responsible for detection of genomic alteration region from the CGH data, and investigation of the relationship between detected alterations and the particular phenotype. The other function is the statistical analysis of the influence of genomic alteration on gene expression profiling. CHESS provides various optional statistical methods for these kinds of analysis, which enables users to choose the proper algorithm for their own data. Additionally, CHESS's detailed visualization module helps users understand massive data easily and intuitively. Finally, CHESS can be used as an essential tool for researchers who study genomic alteration as a molecular marker and characterize its underlying role on downstream mechanism(s) in the pathogenesis of a disease.

## Availability and requirements

Project name: CgHExpreSS

Project homepage: http://biostone.khu.ac.kr/CHESS/

Operating systems: Windows and Linux

Programming language: Java

Other requirements: JRE 6 or higher (Java Runtime Environment)

License: free non-commercial research use license

Any restrictions to use by non-academics: none

## Authors' contributions

ML designed and developed the software and wrote the manuscript. YK supervised the project and revised the manuscript. All authors read and approved the final manuscript.
